# CottonGen: The Community Database for Cotton Genomics, Genetics, and Breeding Research

**DOI:** 10.3390/plants10122805

**Published:** 2021-12-18

**Authors:** Jing Yu, Sook Jung, Chun-Huai Cheng, Taein Lee, Ping Zheng, Katheryn Buble, James Crabb, Jodi Humann, Heidi Hough, Don Jones, J. Todd Campbell, Josh Udall, Dorrie Main

**Affiliations:** 1 Department of Horticulture, Washington State University, Pullman, WA 99164, USA; jing.yu@wsu.edu (J.Y.); sook_jung@wsu.edu (S.J.); chun-huai.cheng@wsu.edu (C.-H.C.); leetaei@wsu.edu (T.L.); ping_zheng@wsu.edu (P.Z.); katheryn.buble@wsu.edu (K.B.); jamescrabb@wsu.edu (J.C.); jhumann@wsu.edu (J.H.); heidi.hough@wsu.edu (H.H.); 2 Cotton Incorporated, Cary, NC 27513, USA; djones@cottoninc.com; 3 The Agricultural Research Service of U.S. Department of Agriculture, Florence, SC 29501, USA; todd.campbell@usda.gov; 4 The Agricultural Research Service of U.S. Department of Agriculture, College Station, TX 77845, USA; Joshua.Udall@usda.gov

**Keywords:** bioinformatics, crop improvement, big data, whole genome sequence, genotype, analysis

## Abstract

Over the last eight years, the volume of whole genome, gene expression, SNP genotyping, and phenotype data generated by the cotton research community has exponentially increased. The efficient utilization/re-utilization of these complex and large datasets for knowledge discovery, translation, and application in crop improvement requires them to be curated, integrated with other types of data, and made available for access and analysis through efficient online search tools. Initiated in 2012, CottonGen is an online community database providing access to integrated peer-reviewed cotton genomic, genetic, and breeding data, and analysis tools. Used by cotton researchers worldwide, and managed by experts with crop-specific knowledge, it continuous to be the logical choice to integrate new data and provide necessary interfaces for information retrieval. The repository in CottonGen contains colleague, gene, genome, genotype, germplasm, map, marker, metabolite, phenotype, publication, QTL, species, transcriptome, and trait data curated by the CottonGen team. The number of data entries housed in CottonGen has increased dramatically, for example, since 2014 there has been an 18-fold increase in genes/mRNAs, a 23-fold increase in whole genomes, and a 372-fold increase in genotype data. New tools include a genetic map viewer, a genome browser, a synteny viewer, a metabolite pathways browser, sequence retrieval, BLAST, and a breeding information management system (BIMS), as well as various search pages for new data types. CottonGen serves as the home to the International Cotton Genome Initiative, managing its elections and serving as a communication and coordination hub for the community. With its extensive curation and integration of data and online tools, CottonGen will continue to facilitate utilization of its critical resources to empower research for cotton crop improvement.

## 1. Introduction

CottonGen serves as the central data repository and analysis resource for the cotton research community, providing access to an integrated and comprehensive online information system to enable basic, translational, and applied cotton research [1]. These activities are supported through funding from industry, government, and academic sources. The first public documentation of CottonGen [2] occurred in 2014, when the database amalgamated and superseded the cotton genome database (CottonDB) [3,4], established in 1995, and the cotton marker database (CMD) [5], established in 2003. CottonGen was expanded to include annotated genome and transcriptome sequences and enhanced with tools for easier data sharing, mining, visualization, and retrieval of cotton research data. In addition, it began hosting the website of the International Cotton Genome Initiative (ICGI), a non-profit organization working to increase knowledge of the structure and function of the cotton genome for the benefit of the global community. The CottonGen database is constructed using the open-source Tripal genome database toolkit [6,7,8], which merges the power of Drupal, a popular web content management system, with that of Chado [9,10], a community-derived database schema for the storage of genomic, genetic, and breeding data [2]. 

Since 2014, there has been an explosion in the volume and type of data generated by the cotton research community. The recent availability of multiple genome assemblies and annotation data from four species in the *Gossypium* genus opened up the opportunity to investigate the evolution and biological basis of various traits of cotton plants, and to share knowledge among major cotton species to improve cultivar performance. To enable the utilization of these big data by the cotton research community, these data were collected, analyzed, and integrated in CottonGen and new tools were developed. Table 1 shows the data currently housed in CottonGen. These data include (i) multiple genome versions of three cultivated species (*G. arboreum*, *G. hirsutum*, *G. barbadense*), one version of the other cultivated species *G. herbaceum*, three new versions of wild species (*G. raimondii*), and eighteen other diploid wild species (a single version of each except two for *G. thurberi* and *G. davidsonii*); (ii) increased gene and RNA-Seq data; (iii) multiple single-nucleotide polymorphism (SNP) arrays; (iv) increased numbers of quantitative trait loci (QTLs) and genetic maps, especially those built with SNPs; (v) SNP genotype data from more breeding programs and projects; (vi) large volumes of phenotype data from multiple germplasm characterizations, breeding trials, and QTL studies; and (vii) a large number of germplasm characterization images from the National Cotton Germplasm Collection (NCGC). In addition to incorporating these new data, we have performed new types of analysis, developed the Cotton Trait Ontology, and standardized terms for cotton trait descriptors that are tightly linked to both the Crop Ontology (CO, vocabulary for crop-related concepts) [11] and the Plant Trait Ontology (TO) [12], and focused on the integration of data across databases and data types. 

This report describes the significantly new and improved data and function added to CottonGen over the last eight years. The value-added efforts undertaken in data analyses, curation, and integration were combined with the development and enhancement of new and existing search interfaces and tools to enable more efficient sharing and reuse of the pivotal data generated by the community. The continued integration of different types of data, as well as the collection and further curation of data, will enable the efficient utilization of these resources for cotton crop improvement. The use of CottonGen continues to increase each year. In 2014, there were 15,666 visits by 7981 researchers from 132 countries, with 90,994 pages served. In the past 12 months (1 September 2020 to 31 August 2021), CottonGen served 357,208 pages to 25,622 researchers from 163 countries with 53,192 visits. Since its inception in 2012, 116,530 researchers have accessed 1,789,713 pages on CottonGen.

## 2. Contents and Functions

### 2.1. Data and Web Interface

The CottonGen interface has been re-designed to provide easier and more intuitive access points to data and tools such as the Major Species Quick Start and Tools Quick Start featured on the homepage (https://www.cottongen.org, accessed on 17 November 2021). The Major Species Quick Start allows researchers to select the interested one among the four cultivated species to view which types of data and tools are available and links to access these data and tools. Similarly, species pages under the ‘Species’ navigation menu provide the same information for cultivated and other species with whole genome sequence data. The Tools Quick Start is organized into genomics, genetics, breeding, and general sections; each section provides links to appropriate pages to access available data, tools, or general information about CottonGen. New features that can quickly familiarize researchers to CottonGen data and functionality include the dynamic data overview page, where researchers can browse the current data types and numbers in CottonGen and access short video tutorials. Tutorials are available for site overview, species pages, the breeding information management system (BIMS) [13], and all the search pages. Below, we describe the currently available data and interfaces, with a focus on new features.

### 2.2. Genomics Data

#### 2.2.1. Whole Genome Sequence Data

CottonGen currently contains fully sequenced cotton genome data from twenty-six Gossypium species: twenty-one diploids and five tetraploids. Among them, four diploid species *G. raimondii*, *G. arboreum*, *G. thurberi*, and *G. davidsonii* (or D5, A2, D1, and D3d-genomes) and two tetraploid species, *G. hirsutum* and *G. barbadense* (or AD1-genome and AD2-genome) have multiple versions of genome sequences (Table 1) produced by different research groups and using different sequencing and assembly technologies. To standardize the genome nomenclature, as well as distinguish the versions of the genome assemblies and annotations from different research groups, CottonGen uses the following naming protocol for new genomes generated by the cotton research community: ‘[Genus] [species] [(genome_group) (optional)] [‘cultivar_name’ (optional)] genome [research_team-sequencing_institute (either one or both)][-special explanation of the assembly (optional)]_v[assembly version number](_a[annotation version number] (if not the same as assembly version number))’. Two examples of this nomenclature for a multiple version of a genome can be seen with the diploid wild species *G. raimondii*: ‘Gossypium raimondii (D5) ‘D5-3’ genome BGI-CGP-draft_v1’ [14] and ‘Gossypium raimondii (D5) genome JGI_v2_a2.1′ [15], reported in the first publication of CottonGen [2].

Forty-four more assemblies have been added in CottonGen (Table 2 and Table 3). They are twenty-eight from diploid species: three new versions of *G. raimondii* [16,17,18]; four versions of *G. arboreum* [18,19,20,21]; one for the other cultivated diploid species *G. herbaceum*; [21], twenty versions of eighteen wild diploid species [17,18,22,23,24,25,26,27] (details in Table 2); and sixteen from tetraploid species—nine versions of *G. hirsutum* [21,28,29,30,31,32,33,34], four versions of *G. barbadense* [31,32,34,35], and one for each of three wild tetraploid species: *G. tomentosum* [34], *G. mustelinum* [34], and *G. darwinii* [34] (Table 3). The predicted genes from these assemblies have been further annotated by the CottonGen team to include homology to cotton proteins from UniProtKB/Swiss-Prot [36,37] and NCBI [38,39] and the proteins of other well annotated or closely related species. In addition, in silico annotation of InterPro [40] protein domains, Gene Ontology (GO) [41] terms, and Kyoto Encyclopedia of Genes and Genomes database (KEGG) [42,43] pathway terms provide information on probable pathways and traits. The CottonGen team also performs synteny analysis to find conserved syntenic regions among all versions of the publicly available Gossypium genomes using MCScanX [44]. Other additional annotations by the CottonGen team includes the alignment of cotton genetic marker sequences and cotton transcripts such as the CottonGen-created RefTrans (www.cottongen.org/data/community_projects/reftrans) to the corresponding genomes. Single nucleotide polymorphisms (SNPs) between the diploid genomes of A and D and those between the tetraploid genomes of AT and DT (subscript T represents tetraploid) were aligned to the JGI version of the *G. raimondii* reference genome [45,46] (https://www.cottongen.org/jbrowse/index.html?data=data%2FGr_JGI_221, accessed on 5 November 2021). 

Assemblies annotated as above can be accessed in their respective genome pages, gene search pages, and BLAST servers, in addition to graphical viewers such as JBrowse [47] and the Tripal Synteny Viewer (https://github.com/tripal/tripal_synview, accessed on 20 September 2021). Each species page provides a summary for the species along with a resource sidebar with hyperlinks to various data and tools for the species, and there is a genome subsection that lists all genome assemblies for the species. Individual genome pages provide downloadable files, including Generic File Format and FASTA formats, for an assembly that includes annotated gene predictions, homology, and positions of repeats and genetic markers including SNPs. Additionally, lists of annotated functional terms and Microsoft Excel files of protein homologs mapped via BLAST+ [48] are available for downloading. These files contain hyperlinks to external databases as well as to CottonGen pages including JBrowse and gene or marker detail pages when applicable. 

The ‘Search Genes and Transcripts’ page allows researchers to search for specific genes and sequences in the above assemblies and transcriptome dataset by species, dataset, gene/transcript name, genomic location, and association with computationally inferred functionality such as GO terms, InterPro domains, and KEGG pathway terms, all in one page. This search interface allows researchers to perform a query such as ‘Return all genes annotated with the word ‘elongation’ between 1.0 and 6.5 Mb on chromosome ‘D12’ of multi-genomes such as ‘*Gossypium hirsutum* (AD1) ‘TM-1’ genome ZJU-improved_v2.1_a1′ and ‘*Gossypium hirsutum* (AD1) ‘TM-1’ genome CRI_v1′. Using the search site, researchers can download the results or proceed to the gene details page within CottonGen. The ‘Customize output’ option allows researchers to customize the result table and the downloadable Excel file to include various functional annotation results. 

**Figure 1:**
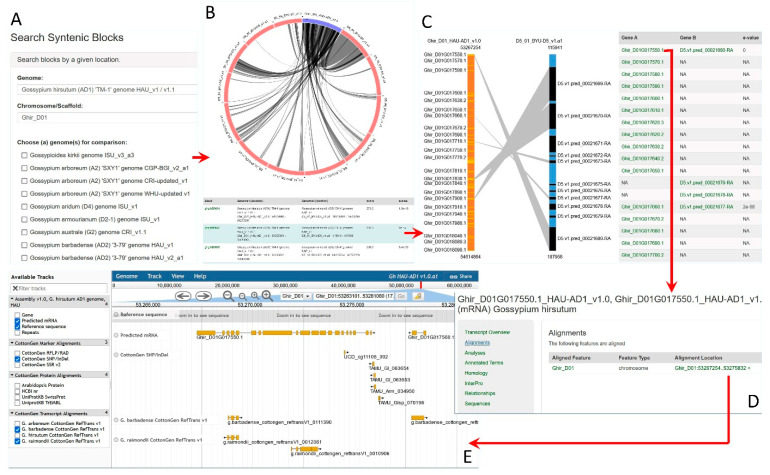
Synteny Viewer in CottonGen. (**A**) Home page of Synteny Viewer allows researchers to choose a chromosome of agenome and multiple genomes for comparison. Researchers can also choose a synteny block ID. (**B**) A circular diagram and a table shows the synteny blocks between a chromosome of a reference genome and all chromosomes of another genome being compared. (**C**) A bar diagram and a table that shows all the genes in a syntenic block. The table displays E-value between the matching genes and the gene names have hyperlinks to the gene detail page. (**D**) A gene detail page with a resource side bar and the hyperlink to JBrowse. (**E**) JBrowse around the mRNA of interest with tracks such as gene, mRNA, SNP and SSR markers.

The gene details page has several links in the resources sidebar to display the sequence and its motif annotations, genome alignments, and homologies to sequences of other species in CottonGen and other databases. The alignment details provide links to view a gene in JBrowse. The new synteny section lists orthologs and paralogs in other genomes discovered by synteny analyses and provides hyperlinks to the gene pages and Synteny Viewer. Via the ‘Synteny Viewer’ page (Figure 1A), accessible from the ‘Tools’ navigation menu, researchers can choose a scaffold/chromosome of one reference genome and choose multiple other genomes for comparison. The viewer then displays a clickable circular image as well as a table showing all the syntenic blocks between the genomes (Figure 1B). Choosing one block either from the image or the table leads to a page where all the syntenic genes within the block are shown with the Expect value (E-value) of their homology (Figure 1C). Gene names are linked to a specific gene page (Figure 1D) where orthologs/paralogs in other genomes are available among other information about the genes, such as associated functions and genomic location with a link to JBrowse (Figure 1E). Conserved synteny data made available in CottonGen thus allows researchers starting with one cotton genome to explore genes, anchored trait loci, and genetic markers within orthologous regions of another cotton genome. Using JBrowse, researchers can view all the genomic features aligned to the genome, including gene models, predicted mRNAs, repeats, SNPs, and other genetic markers, and genes from other model plant species. CottonGen uses the Tripal BLAST module (http://tripal.info/extensions/modules/tripal-blast-analysis, accessed on 20 September 2021), replacing the old BLAST and batch BLAST tools. The new BLAST enables results to link to the genome scaffolds in JBrowse and to the gene/transcript detail pages in CottonGen and in NCBI. Predicted genes from whole genome sequences were employed in the construction of CottonCyc (metabolic pathway) databases [49] using PathwayTools [50].

#### 2.2.2. Transcriptome Data

The CottonGen team combines peer-reviewed published RNA-Seq and EST data sets to create a reference transcriptome (RefTrans) for Gossypium species and provides the putative gene function identified by homology to known proteins. The RNA-Seq sequences from peer-reviewed publications were downloaded from the NCBI Short Read Archive (SRA) and subject to quality control using Trimmomatic (v0.32, default parameters, [51]) and custom Perl scripts. The remaining RNA-Seq reads were assembled de novo with Trinity (v2.6.6) [52] using default assembly parameters and a minimum coding length of 200 bases. Quality control of the ESTs included vector sequence screening (UniVec_Core, ftp://ftp.ncbi.nih.gov/pub/UniVec/, accessed on 17 August 2017) using cross_match [53], the removal of tRNA/rRNA/snRNA sequences identified using tblastx [54], and Poly-A tail trimming. The filtered ESTs were assembled using the CAP3 program (P−90) [55]. Bowtie (v 2.3.3) [56] was applied to multi-map the RNA-Seq reads and ESTs back to the assembled contigs and singlets. The contigs and singlets were clustered into genes using CH-HIT (v4.6.4) [57] and Corset (v1.0.7) [58] with default parameters. The longest isoform greater than 500 nt was selected to represent each Corset cluster and create the RefTrans sequences. The RefTrans sequences are functionally characterized by pairwise comparison using the BLASTX algorithm against the Swiss-Prot and TrEMBL protein databases. Information on the top ten matches with an E-value of ≤ 1 × 10^−6^ are recorded and stored in CottonGen together with the RefTrans sequences. InterPro domains and Gene Ontology assignments were made using InterProScan [59] at the EBI (European Bioinformatics Institute, https://www.ebi.ac.uk, accessed on 17 August 2017) through Blast2GO [60]. Transcriptomes and their associated annotation are available to download in the transcriptome page that can be accessed from each species page, to search and download in the ‘Search Genes and Transcripts’ page, to view on the genome in JBrowse, and to perform similarity searches in the BLAST server. Current CottonGen RefTrans datasets (v1.0) include the four genome sequenced species: *G. raimondii*, *G. arboreum*, *G. hirsutum*, and *G. barbadense*. 

#### 2.2.3. NCBI Genes

CottonGen periodically downloads Gossypium sequences from the NCBI. All sequences are then parsed for gene, mRNA, CDS, 5′UTR, and 3′UTR features and imported to CottonGen. As with predicted genes from whole genome sequences, genes parsed from NCBI have been further annotated by homology to genes in other species, InterPro protein domains, GO terms, and KEGG pathway terms. The distinct gene names in Gossypium are stored separately in the database to build a community-driven gene database for cotton. Each gene, unique in the Gossypium genus, is currently linked to all the NCBI genes from various species and will serve as a base entity to be linked to other associated data, such as predicted genes from whole genome sequences, QTL, genetic markers, and mutant phenotypes as annotation progresses. All genes and mRNAs that are parsed out from NCBI sequences are searchable in the gene search page. 

### 2.3. Genetics Data

#### 2.3.1. Genetic Marker and SNP Array Data

CottonGen contains detailed data on more than 500,000 genetic markers (Table 1) used in genetic map development, genetic diversity studies, genome wide association studies, and SNP array development. Marker annotations include marker aliases, source germplasm, source description, primer sequences, polymerase chain reaction conditions, literature references, and map position where available. For SNPs, the marker details also list the SNP marker name, SNP ID name in SNP array, alleles, flanking sequences, and probes. SNP marker data available in CottonGen includes those from array development projects such as the TAMU CottonSNP63K Array [61] and the NAU80K Array [62]. The SNP array data are available to download in Microsoft Excel format, to view in JBrowse, and to query in the ‘Marker Search’ page. The ‘Marker Search’ options now have a ‘SNP Marker Search’ tab in addition to the ‘Marker Search’, ‘Marker Source’, ‘Nearby Loci’, ‘Nearby QTL’, and ‘Between Markers’ tabs. The search filter in the ‘Marker Search’ page includes marker name, marker type, the species from which the marker is developed, the species to which the marker is mapped, and map position in the genetic map and genome. Filtering by trait name is a new feature that allows researchers to search for markers that are near and/or within QTLs using the associated trait name. The table in the results page shows the marker name, alias, marker type, species, genetic map location, and genome location. The downloaded file contains the same information plus the citation. The ‘SNP Marker Search’ tab is designed so that researchers can filter using array information as well as SNP name and genomic location. The results table is also specific for SNP, with alleles, SNP array information, genome location, and flanking sequences. In both search pages, researchers can upload a file of marker names for querying. Other search interface tabs, ‘Nearby Loci’ and ‘Nearby QTL’, enable researchers to find markers near a targeted marker locus or QTL, and ‘Between Markers’ allows researchers to pull out all markers between the two specific markers where available on any genetic maps.

#### 2.3.2. Genetic Maps and QTLs 

With a continuous effort to curate peer-reviewed published data as it becomes available, CottonGen now contains 121 genetic maps across multiple Gossypium species, consisting of 130,533 molecular/morphological/gene marker loci and 6772 quantitative trait loci (QTLs). The ‘Map Data Summary’ (https://www.cottongen.org/find/featuremap/summary, accessed on 29 October 2021) is found under ‘Search’ and ‘Search Map’, and it dynamically provides general information about maps in CottonGen with a link to the home page of each map and the parent(s) of the mapping population. The data associated with the genetic maps include the mapped positions of molecular markers, QTLs, and heritable phenotypic markers, environments, as well as mapping population(s) and associated publication(s). The ‘Search QTLs’ link in the ‘Search’ main menu allows researchers to find QTLs and/or MTLs (Mendelian trait loci) by any combination of trait category, trait name, and published symbol or label.

CottonGen now uses a new graphic interface, MapViewer [63] (https://www.cottongen.org/MapViewer, accessed on 29 October 2021), to display genetic maps. Selecting ‘Tools’ then ‘MapViewer’ allows researchers to view and compare maps from different populations and species, facilitating information transfer from well-studied to less-studied species. These comparisons are very useful due to the well-conserved synteny among the genomes of Gossypium species. While the functionality of MapViewer is like CMap [64], a commonly used tool in biological databases, MapViewer is more heavily integrated with other pages, such as the map, marker, QTL, and genome pages. In addition, MapViewer allows researchers to zoom into specific regions of a linkage group, choose the types of markers to be displayed, and change the color of the markers that are displayed. 

#### 2.3.3. Genotypic and Phenotypic Diversity Data 

**Figure 2:**
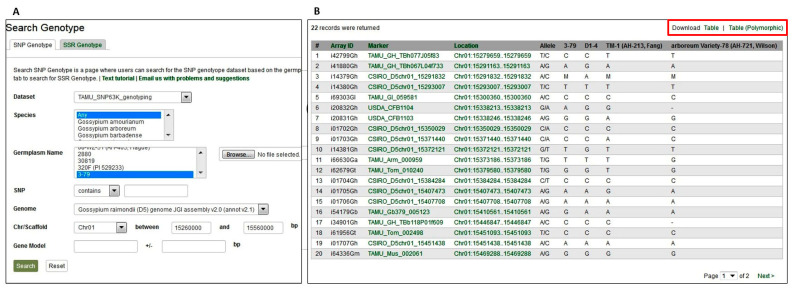
SNP Genotype search page in CottonGen. (**A**. left) Researchers can search SNP genotype data by dataset name, species, germplasm name, SNP name, genome location, and/or gene name. Researchers can also upload a file with a list of germplasm names. (**B**. right) Search result table that shows SNP name, genome location, allele, and the genotype data of all the germplasm chosen in the order of SNP location in the genome. The red square highlights the options to download the genotype for all the markers displayed in the result page or the genotype data that are polymorphic in the germplasm set chosen.

CottonGen contains over 25 million genotypic data points and over half a million phenotypic diversity data points (Table 1) from published genetic studies, breeding trials, and germplasm trait evaluations. Clicking on ‘Search’ then ‘Search Genotype’ gives researchers the ability to search the 7 and 4 genotypic datasets available for SSRs and SNPs, respectively. In the ‘SNP Genotype’ search tab, researchers can filter the results by dataset name, species, germplasm name, SNP name, genomic location, and/or gene name (Figure 2A). Researchers can also upload a file with germplasm names. This filtering allows researchers to perform tailored querying, such as finding SNP polymorphisms around a gene of interest in a chosen set of germplasm. The results table provides the SNP name, genomic location, allele, and genotypic data of all the germplasm chosen in the order of the SNP location in the genome, so that researchers can view the genotype of each germplasm along the chromosome (Figure 2B). Researchers can download the genotypes for all markers displayed in the results page or the genotypes for only the markers that are polymorphic within the germplasm set chosen (Figure 2B). Phenotypic diversities in CottonGen includes breeders’ trial evaluation data from the US Regional Breeders Testing Network (RBTN, https://rbtn.cottoninc.com/about/, accessed on 15 November 2021); QTL related trait evaluation data collected from published journal articles; and germplasm evaluation data from the US National Cotton Germplasm Collection (NCGC), the China Cotton Germplasm Collection (CN_COT), and the Uzbekistan Cotton Germplasm Collection (UZ_COT). The ‘Search Trait Evaluation’ page under the ‘Search’ menu provides options to query either qualitative or quantitative traits. In each tab, researchers can filter the data by trait cut-off values of up to three trait descriptors. The results table and downloadable file have the germplasm name, species, the trait values chosen, and the dataset name. The germplasm and the dataset name in the results table are linked to the detail page where other associated data can be accessed. The germplasm page has a resource sidebar for genotypic and phenotypic data, as well as an overview and associated images, where the data can be viewed and downloaded.

#### 2.3.4. Cotton Trait Ontology

Using controlled vocabularies to standardize the agronomic phenotypic descriptors is one of the essential steps in data annotation, integration, analysis, interpolation, and sharing across projects, species, and databases, all of which can expedite gene discovery. For this purpose, the CottonGen team, together with cotton specialists, developed the first set of standardized ontologies that includes phenotypic trait information found in cotton species—Cotton Trait Ontology (https://www.cottongen.org/data/trait_ontology, accessed on 15 November 2021). Researchers can access ‘Data’ then ’Cotton Trait Ontology’ to see the set of standardized and structured vocabularies for cotton traits and trait descriptors, which consolidates terms from cotton trial evaluations (RBTN and NCVT, National Cotton Variety Test), cotton germplasm evaluations from three countries, and QTL-trait association data obtained from over one hundred peer-reviewed publications. In order to integrate information across databases and data types, the Cotton Trait Ontology is connected to the larger vocabulary and database community via Crop Ontology (CO) [11] and Plant Trait Ontology (TO) [12]. Each of the terms in the Cotton Trait Ontology is either an existing TO term or will be added to the TO, and all of these terms have been submitted to Crop Ontology (http://www.cropontology.org, CO_358, accessed on 15 August 2021). The current Cotton Trait Ontology contains 223 traits of 12 trait classes associated with 303 trait descriptors and will continue to be updated and validated as new data is imported to CottonGen. 

### 2.4. Breeding Data and Breeding Information Management System

**Figure 3:**
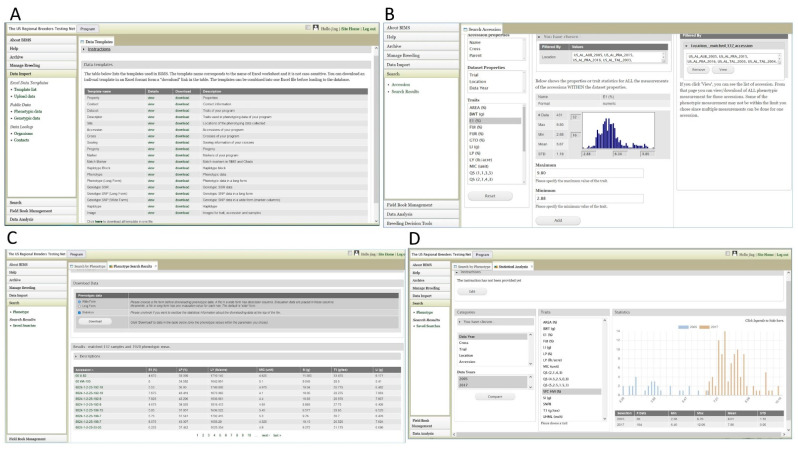
CottonGen BIMS. (**A**) ‘Template List’ subsection in ‘Data Import’ section provides downloadable templates for researchers to enter various breeding data. (**B**) ‘Search’ section allows researchers to search and save the list of accessions using any combination of properties and trait cut of values: accessions name, trial, location, cross, data year, and trait values. The middle section shows the statistical information on the filtered dataset for the trait chosen and the right section shows the number of accessions filtered so far. (**C**) A page with the search result table. Researchers can add more columns in the table using ‘Column options’ and save/download the result table. (**D**) ‘Data Analysis’ section that allows researchers to choose multiple datasets, using the categories or saved accession lists and compare the trait statistics between the datasets.

A new secure and comprehensive online breeding information management system (BIMS), developed for the generic Tripal Database Platform [13], http://tripal.info/extensions/modules/tripal-bims, accessed on 2 April 2021), is used in CottonGen, which allows individual breeders to integrate their data with public genomic and genetic data and at the same time have complete control of their own breeding data and access to tools such as data import/export, data analysis, and a data archive. BIMS also allows researchers to compare and query historical germplasm characterization and evaluation data to select parents, donors, and recipients for crossing. BIMS incorporates the use of an Android app called Field Book [65], an open-source software for smartphones and tablets, which enables breeders to replace hard-copy field books for recording notes, thus alleviating the possibility of transcription errors while providing faster access to the collected data. The use of Field Book and BIMS promotes the development and implementation of standard trait descriptors and the collection of metadata. Current data in CottonGen BIMS include phenotypic, genotypic, germplasm, and pedigree data from both public and private breeding projects. BIMS contains publicly available evaluation data from breeding trials (RBTN), germplasm collections (NCGC, CN_COT, UZ_COT), and a few QTL mapping populations [66,67]. These data are free to access, query, and download through BIMS. In BIMS, an accordion menu in each page provides quick access to various functionalities. The ‘Data Import’ section provides data templates for researchers to enter their data and upload the data themselves (Figure 3A). The ‘Search’ section allows researchers to search and save the list of germplasm individuals (‘accessions’) using any combination of properties and trait cut-off values: name, trial, location, cross, parent, and trait values (Figure 3B). When a filter is applied to chosen accessions, the rightmost section shows the number of accessions available in the filtered dataset. When a trait descriptor is chosen as a filter, the middle section shows a histogram along with the statistical values, such as maximum, minimum, mean, and standard deviation, to visualize the distribution of data points within the chosen dataset (Figure 3B). The list of the accession names chosen can be viewed and downloaded in a table with an option of adding more data about the accessions such as location, data year, and trait values (Figure 3C). The list of accessions can be saved in user accounts and can be used to retrieve any data associated with the accessions. The ‘Data Analysis’ section allows researchers to choose multiple datasets, using the categories or saved accession lists, and compare the trait statistics between the multiple datasets (Figure 3D). Researchers can also narrow down the datasets in the ‘Search’ section and then compare the trait statistics among multiple sub-datasets. For example, this analysis function allows researchers to compare various traits of multiple sets, such as accessions evaluated in the years of 2005, 2010, and 2017 for a dataset from a specific cross.

### 2.5. Community Resources

On the CottonGen home page, there is a ‘News and Events’ section for news of significant merit, including publications, availability of larger datasets, or upcoming community events. CottonGen continues to house the resources for ICGI (the International Cotton Genome Initiative) under the ‘ICGI’ navigation menu, including maintaining the ICGI membership database and hosting information for ICGI elections that happen in odd-numbered years, and for the ICGI international research conferences that take place in even-numbered years. Functionality of the ICGI website was enhanced to include online conference registration, abstract submission, automatic distributions to the leaders of associated workgroups to view and select oral presentations, and election ballot distribution to valid members to vote and automatically generate an election report. Under the ‘Data’ navigation menu, there are ‘Community Projects’ and ‘Community Archives’ that provide information on special research projects that have multi-institutions/research groups involved or standardized information and data for the nationwide cotton community (such as ‘TAMU CottonSNP63K Array’ and ‘USDA-ARS NCGC Characterization’), which consists of general information, developed data, publications, etc., of the project; the special archives, meanwhile, contain historical data such as the history of the Cotton Improvement Conference and scanned images of bulletins that are difficult to obtain from online or public library sources (such as ‘Cotton Improvement: 1948 to 2018’). The General navigation menu provides information about CottonGen, including a brief description of CottonGen, recently completed as well as planned work, and presentations. Several mailing lists, in addition to the CottonGen mailing list, are available to serve the community with information for specific interests or purposes, and the archives can be viewed through the message board sites. The ‘Help’ navigation menu provides a CottonGen user manual and frequently asked question pages for both CottonGen and ICGI. 

## 3. Data Collection, Submission and Download

Large datasets, such as whole genome sequences, SNP arrays, germplasm collection characterizations, and reginal breeders trial data were either contributed by research groups or obtained from the project original repository website under agreement. Smaller datasets, such as Genetic Maps, Markers, and QTLs were collected from publications. The publication data and NCBI cotton sequence data were periodically collected by the Tripal Publication Importer and the Tripal GenBank Parser. While the CottonGen team actively curated data from publications, the ‘Data Submission’ page under the ‘Data’ navigation menu provides links to templatesfrequently used for marker, genetic map, QTL, genotype, and phenotype data submissions. We encourage authors to officially archive their datasets by submitting their data at the time of manuscript submission. The ‘Data Contributors’ page lists people who contributed data to CottonGen (also CottonDB and CMD before they were consolidated into CottonGen) with links to whole genome sequences and special community-archived information when available. On the ‘Data Download’ page, the section for ‘Whole Genome Sequence Data’ contains sub-pages under ‘Diploid Genomes’ or ‘Tetraploid Genomes’. Each sub-page provides a side-by-side list of assembly and annotation information, which allows a user to easily compare and download data from the original sequencing project and annotation data that was added by the CottonGen team. Furthermore, most other types of data can be downloaded through the search interfaces, but for researchers’ convenience, bulked marker sequences in FASTA format and marker primers in Excel format are provided under the ‘Marker Data’ subsection on the ‘Data Download’ page.

## 4. Concluding Remarks and Future Direction

To facilitate the utilization of cotton research data in basic discovery, translation, and crop improvement, CottonGen, over the last decade, has focused on integrating new whole genomic data with transcriptomic, genetic map, genetic marker, trait locus, phenotypic, and genotypic data. To accommodate the data mining needs that came with these new types and large volumes of data, various web interfaces were developed by the CottonGen team, such as MegaSearch [68], MapViewer [63], BIMS [13], Chado Loader, Chado Data Display, and Chado Search modules [69], or Tripal modules that other database teams developed such as the Synteny Viewer and Tripal BLAST [8]. The open-source database platform Tripal allows database teams to meet emerging demands for storage, querying, and the display of new data types more efficiently and quickly. During the last eight years, major advances were made in curating data for CottonGen with more ontologies developed and incorporated in data query pages facilitating data sharing across different data types, species, and databases. This work benefits biological database developers, as well as cotton researchers and breeders, as Tripal extension modules are shared across databases.

Future efforts will include the further development MapViewer to integrate genome data and genetic maps, providing enhanced querying interfaces, expanding the analysis capabilities in BIMS through access to additional functionality, GWAS analysis capability, and providing access to high performance computing. Further effort will also include the curation of new data types such as pan-genome data, epigenome data, expression data, phenomics data, as the addition of increasing volume of current data types in CottonGen. 

Use of CottonGen, the community database resource for cotton genomics, genetics, and breeding research, has continued to grow. Between 16 August 2013 and 15 August 2021, CottonGen had 247,717 visits by 109,663 unique visitors from 194 countries, who accessed 1,688,706 pages. The CottonGen database is part of AgBioData [70] (https://www.agbiodata.org, accessed on 10 September 2021), a consortium working to improve the standards and sustainability of agricultural genomics, genetics, and breeding databases and further enable agricultural science.

## Figures and Tables

**Table 1:** Comparison of number of CottonGen entries between 15 August 2013 and 31 August 2021 by data type.

Type	By 8/14/13	By 8/31/21	Data Details by 31 August 2021
Genome	*G.raimondii (2*)	46 (30 diploids and 16 tetraploids)	Whole genome assemblies and annotations of 30 diploid species: *G.anomalum (1)*, *G.arboreum (4)*, *G.aridum (1)*, *G.armourianum (1)*, *G.australe (1)*, *G.davidsonii (2)*, *G.gossypioides(1)*, *G.harknessii (1)*, *G.herbaceum (1)*, *G.kirkii (1)*, *G.klotzschianum (1)*, *G.laxum (1)*, *G.lobatum (1)*, *G.longicalyx (1)*, *G.raimondii (5)*, *G.rotundifolium (1)*, *G.schwendimanii (1)*, *G.stocksii (1)*, *G.thurberi (2)*, *G.trilobum (1)*, *G.turneri (1);* and 5 tetraploid species: *G.hirsutum (9)*, *G.barbadense (4)*, *G.tomentosum (1)*, *G.mustelinum (1)*, *G.darwinii (1)*
Gene and mRNA	119,971 genes	1,874,940 genes and 2,528,191 mRNAs	Genes and mRNAs from whole genome assemblies and parsed from NCBI nucleotide sequences
Transcript	149,916	214,180 RefTrans	RefTrans for *G.hirsutum*, *G.barbadense*, *G.arboreum*, *G. raimondii*
Marker	26,089	587,004	Including 459,825 SNPs (TAMU63K and NAU80K arrays, and other SNPs), 109,848 SSRs
Map	49	115	130,533 loci from 110 genetic maps, 2 consensus maps, 2 bin maps, and 1 silico map
QTL	988	6772	Including 4178 quality traits, 1547 agronomical trait, 273 biotic stress traits, and 189 biochemical traits
Species	50	85	Including the 4 cultivated species, 53 wild species, and 28 cross or lab made diploid, tetraploid, and hexaploidy hybrids
Germplasm	14,959	19,827	Including collection and sub-collections from US-NCGC, US-GRIN, China, and Uzbekistan
Phenotype data	118,302	539,975	Phenotypic scores from the US regional breeder’s tests; the trait evaluations from US, Uzbekistan, and China germplasm collections; and the data collected from various QTL studies
Genotype data	68,640	25,532,891	SNP genotype data from 25,213,321 measurements using 71,424 markers, SSR genotype data from 319,570 measurements using 2825 markers
Image	0	45,211	Including 44,998 NCGC digital characterizations
Publication	10,731	16,066	Including journal articles, conference proceedings, patents, book chapters, and theses/dissertations
Library	181	181	Including 135 cDNA, 41 genomic DNA, 2 SNP chip, and 2 unassigned libraries

**Table 2:** List of 30 diploid genome sequences available in CottonGen (by 31 August 2021).

Genome Sequence Name	Germplasm Type	Pub Year (Ref.)
*Gossypium raimondii* (D5) ‘D5-3’ genome CGP-BGI_v1	wild	2012 [14]
*Gossypium raimondii* (D5) genome JGI_v2_a2.1	wild	2012 [15]
*Gossypium raimondii* (D5) ‘D5-4’ genome NSF_v1	wild	2019 [16]
*Gossypium raimondii* (D5) ‘D5-8’ genome ISU_v1	wild	2019 [17]
*Gossypium raimondii* (D5) ‘D502’ genome HAU_v1	wild	2021 [18]
*Gossypium arboreum* (A2) ‘SXY1’ genome CGP-BGI_v2_a1	cultivar	2014 [19]
*Gossypium arboreum* (A2) ‘SXY1’ genome CRI-updated_v1	cultivar	2018 [20]
*Gossypium arboreum* (A2) ‘SXY1’ genome WHU-updated_v1	cultivar	2020 [21]
*Gossypium arboreum* (A2) ‘SXY1’ genome HAU_v1	cultivar	2021 [18]
*Gossypium herbaceum* (A1) ‘Mutema’ genome WHU_v1	cultivar	2020 [21]
*Gossypium anomalum* (B1) genome NSF_v1	wild	2021 [22]
*Gossypium thurberi* (D1-35) genome ISU_v1	wild	2019 [17]
*Gossypium thurberi* (D1-5) genome CRI_v1	wild	2021 [23]
*Gossypium armourianum* (D2-1) genome ISU_v1	wild	2019 [17]
*Gossypium harknessii* (D2-2) genome ISU_v1	wild	2019 [17]
*Gossypium davidsonii* (D3d-27) genome ISU_v1	wild	2019 [17]
*Gossypium davidsonii* (D3d-8) genome CRI_v1	wild	2021 [23]
*Gossypium klotzschianum* (D3-k) genome ISU_v1	wild	2019 [17]
*Gossypium aridum* (D4) genome ISU_v1	wild	2019 [17]
*Gossypium gossypioides* (D6) genome ISU_v1	wild	2019 [17]
*Gossypium lobatum* (D7) genome ISU_v1	wild	2019 [17]
*Gossypium trilobum* (D8) genome ISU_v1	wild	2019 [17]
*Gossypium laxum* (D9) genome ISU_v1	wild	2019 [17]
*Gossypium turneri* (D10) genome NSF_v1_a2	wild	2019 [17]
*Gossypium schwendimanii* (D11) genome ISU_v1	wild	2019 [17]
*Gossypium stocksii* (E1) genome NSF_v1	wild	2021 [24]
*Gossypium longicalyx* (F1) genome NSF_v1	wild	2020 [25]
*Gossypium australe* (G2) genome CRI_v1.1	wild	2019 [26]
*Gossypium rotundifolium* (K12) ‘Grot K201’ genome HAU_v1	wild	2021 [18]
*Gossypioides kirkii* genome ISU_v3	wild	2019 [27]

**Table 3:** List of 16 tetraploid genome sequences available in CottonGen (by 31 August 2021).

Genome Sequence Name	Germplasm Type	Pub Year (ref.)
*Gossypium hirsutum* (AD1) ‘TM-1’ genome CGP-BGI_v1	cultivar	2015 [28]
*Gossypium hirsutum* (AD1) ‘TM-1’ genome NAU-NBI_v1.1	cultivar	2015 [29]
*Gossypium hirsutum* (AD1) ‘TM-1’ Genome UTX-JGI-interim-release_v1.1	cultivar	2017 [30]
*Gossypium hirsutum* (AD1) ‘TM-1’ genome HAU_v1	cultivar	2018 [31]
*Gossypium hirsutum* (AD1) ‘TM-1’ genome ZJU-improved v2.1_a1	cultivar	2019 [32]
*Gossypium hirsutum* (AD1) ‘TM-1’ genome CRI_v1	cultivar	2019 [33]
*Gossypium hirsutum* (AD1) ‘ZM24’ genome CRI_v1	cultivar	2019 [33]
*Gossypium hirsutum* (AD1) ‘TM-1’ genome WHU_v1	cultivar	2020 [21]
*Gossypium hirsutum* (AD1) ‘TM-1’ genome UTX_v2.1	cultivar	2020 [34]
*Gossypium barbadense* (AD2) ‘3-79’ genome HAU_v1	cultivar	2015 [35]
*Gossypium barbadense* (AD2) ‘3-79’genome HAU_v2_a1	cultivar	2018 [31]
*Gossypium barbadense* (AD2) ‘H7124’ genome ZJU_v1.1_a1	cultivar	2019 [32]
*Gossypium barbadense* (AD2) ‘3-79’ genome HGS_v1.1	cultivar	2020 [34]
*Gossypium tomentosum* (AD3) gnome HGS_v1.1	wild	2020 [34]
*Gossypium mustelinum* (AD4) genome JGI_v1.1	wild	2020 [34]
*Gossypium darwinii* (AD5) genome HGS_v1.1	wild	2020 [34]

## Data Availability

All data on CottonGen.org (accessed on 17 November 2021) is publicly available.
